# Statins do not increase Markers of Cerebral Angiopathies in patients with Cardioembolic Stroke

**DOI:** 10.1038/s41598-018-20055-3

**Published:** 2018-01-24

**Authors:** Joan Martí-Fàbregas, Santiago Medrano-Martorell, Elisa Merino, Luis Prats-Sánchez, Rebeca Marín, Raquel Delgado-Mederos, Pol Camps-Renom, Alejandro Martínez-Domeño, Manuel Gómez-Choco, Lidia Lara, Ignacio Casado-Naranjo, David Cánovas, Maria José Torres, Marimar Freijo, Ana Calleja, Yolanda Bravo, Dolores Cocho, Ana Rodríguez-Campello, Beatriz Zandio, Blanca Fuentes, Alicia de Felipe, Laura Llull, José Maestre, María Hernández, Moisès Garcés, Ana Maria De Arce-Borda, Ernest Palomeras, Manuel Rodríguez-Yáñez, Inma Díaz-Maroto, Marta Serrano, Jéssica Fernández-Domínguez, Jordi Sanahuja, Francisco Purroy, Marialuisa Zedde, Jordi Delgado-Mengual, Ignasi Gich

**Affiliations:** 1 Department of Neurology, Hospital de la Santa Creu i Sant Pau, Biomedical Research Institute, Barcelona, Spain; 2 Department of Radiology, Hospital del Mar- Parc de Salut Mar, Barcelona, Spain; 3 Hospital Germans Trias i Pujol, Unitat RM IDI, Badalona, Spain; 4 Department of Epidemiology, Hospital de la Santa Creu i Sant Pau, Biomedical Research Institute, Barcelona, Spain; 5 Department of Neurology, Hospital de Sant Joan Despí Moises Broggi, Sant Joan Despí, Spain; 6 Department of Neurology, Hospital de León, León, Spain; 7 Department of Neurology, Hospital San Pedro de Alcántara, Cáceres, Spain; 8 Department of Neurology, Hospital Parc Taulí, Sabadell, Spain; 9 Department of Neurology, Hospital Son Espases, Palma de Mallorca, Spain; 10 Department of Neurology, Hospital de Basurto, Bilbao, Spain; 11 Department of Neurology, Hospital de Valladolid, Valladolid, Spain; 12 Department of Neurology, Hospital de Burgos, Burgos, Spain; 13 Department of Neurology, Hospital de Granollers, Granollers, Spain; 14 Department of Neurology, Hospital del Mar, Barcelona, Spain; 15 Department of Neurology, Hospital de Navarra, Pamplona, Spain; 16 Department of Neurology, Hospital Universitario La Paz, Instituto de Investigación IdiPaz, Madrid, Spain; 17 Department of Neurology, Hospital Ramón y Cajal, Madrid, Spain; 18 Department of Neurology, Hospital Clínic, Barcelona, Spain; 19 Department of Neurology, Hospital Virgen de las Nieves, Granada, Spain; 20 Department of Neurology, Hospital Germans Trias i Pujol, Badalona, Spain; 21 Department of Neurology, Hospital Verge de la Cinta, Tortosa, Spain; 22 Department of Neurology, Hospital de Donostia, Donostia, Spain; 23 Department of Neurology, Hospital de Mataró, Mataró, Spain; 24 Department of Neurology, Hospital Santiago de Compostela, Santiago de Compostela, Spain; 25 Department of Neurology, Hospital de Albacete, Albacete, Spain; 26 Department of Neurology, Hospital de La Rioja, Logroño, Spain; 27 Department of Neurology, Centro Médico Asturias, Oviedo, Spain; 28 Department of Neurology, Hospital Arnau de Vilanova, Lleida, Spain; 29 Department of Neurology, Arcispedale Santa Maria Nuova IRCCS, Reggio Emilia, Italy; 30 Port d’Informació Científica, Institut de Física d’Altes Energies, Campus UAB, Cerdanyola del Vallès, Spain

## Abstract

We investigated whether pre-treatment with statins is associated with surrogate markers of amyloid and hypertensive angiopathies in patients who need to start long-term oral anticoagulation therapy. A prospective multicenter study of patients naive for oral anticoagulants, who had an acute cardioembolic stroke. MRI was performed at admission to evaluate microbleeds, leukoaraiosis and superficial siderosis. We collected data on the specific statin compound, the dose and the statin intensity. We performed bivariate analyses and a logistic regression to investigate variables associated with microbleeds. We studied 470 patients (age 77.5 ± 6.4 years, 43.7% were men), and 193 (41.1%) of them received prior treatment with a statin. Microbleeds were detected in 140 (29.8%), leukoaraiosis in 388 (82.5%) and superficial siderosis in 20 (4.3%) patients. The presence of microbleeds, leukoaraiosis or superficial siderosis was not related to pre-treatment with statins. Microbleeds were more frequent in patients with prior intracerebral hemorrhage (OR 9.7, 95% CI 1.06–90.9) and in those pre-treated antiplatelets (OR 1.66, 95% CI 1.09–2.53). Prior treatment with statins was not associated with markers of bleeding-prone cerebral angiopathies in patients with cardioembolic stroke. Therefore, previous statin treatment should not influence the decision to initiate or withhold oral anticoagulation if these neuroimaging markers are detected.

## Introduction

It is common to prescribe statins for old patients. In a systematic review, up to 48% of patients who had an ischemic stroke were receiving statins prior to the stroke^1^. Statins are prescribed to prevent untoward vascular events, including ischemic stroke^2^. However, statins may increase the risk of intracerebral hemorrhage (ICH), due to the lowering of cholesterol levels and their antithrombotic properties^3^. Also, it is controversial whether or not statin therapy is linked to cerebral amyloid angiopathy (CAA) by increasing the likelihood of microbleeds (MB) and lobar hemorrhages^4–10^. Finally, the influence of the specific statin compound, the statin dose and the statin potency on the hemorrhagic risk has not been evaluated.

The fear of major bleeding, including intracranial hemorrhage, is the main reason why oral anticoagulants (OA) are underused in the elderly. Thus, the study of factors that contribute to the risk of hemorrhagic complications in long-term anticoagulation candidates is of enormous importance. It is known that cerebral hemorrhagic-prone amyloid and hypertensive angiopathies increase the risk of ICH in older patients. Additionally, drugs with antithrombotic properties might increase ICH risk in patients with these angiopathies^11–13^. Consequently, the combination of statins and antiplatelet or OA drugs, might lead to excessive hemorrhagiing.

In a prospective study, our aim was to investigate whether prior statin therapy increases the risk of MB, cortical superficial siderosis (SS) and leukoaraiosis (LK) in patients with cardioembolic stroke who did not take OA and who were candidates to begin indefinite treatment with OA. Our hope was that our study would provide guidance regarding the use of statins in patients who have suffered a cardioembolic stroke and in whom OA are to be prescribed.

## Material and Methods

We studied patients with transient ischemic attack (TIA) or cerebral infarct attributed to cardiac embolism after a complete etiologic work-up and who were considered candidates for indefinite OA treatment. For most patients, atrial fibrillation (AF) was the embolic source, but patients with other high-risk cardioembolic sources were also permitted. All of the patients were naïve to OA. The patients were participants in the HERO study. The HERO study (risk of intracranial HEmorrhage predicted by Resonance in patients receiving Oral anticoagulants, ClinicalTrials.gov Identifier: NCT02238470), a currently ongoing multicenter observational study. Briefly, the aim of the study is to assess whether the risk of intracranial hemorrhage (intracerebral and extracerebral) in patients with cardioembolic stroke who start a long-term treatment with OA is influenced by the detection of surrogate markers of hemorrhagic-prone angiopathies in the MRI. All methods were carried out in accordance with relevant guidelines and regulations. All experimental protocols were approved by the Ethics Committee at Hospital de la Santa Creu i Sant Pau and also at each center. Informed written consent was required for study participation. The patient or a legal representative signed the written consent to participate.

### Patients

Inclusion criteria were: 1) age equal or above 65 years; 2) TIA or acute ischemic infarct; 3) the patient is considered a good candidate to receive long-term OA for the secondary prevention of ischemic stroke; 4) the patient is a new user of any OA (vitamin K antagonist or direct OA); 5) the consent to participate is signed before performing MR; 6) MR is performed within 1 month of the index ischemic stroke.

Exclusion criteria were: 1) Primary prevention of cardiac embolism; 2) Indication of OA other than the prevention of cardiac embolism; 3) Absolute contraindication to OA treatment; 4) Uncontrolled high blood pressure and hypertensive crisis; 5) Dementia; 6) Life expectancy less than 1 year; 7) Any contraindication to perform an MR.

The following clinical variables were recorded for each patient: 1) Demographic data (age and gender); 2) Traditional vascular risk factors (previous cerebral infarct and TIA, previous intracerebral hemorrhage, hypertension, diabetes mellitus, hypercholesterolemia, hypertriglicerydemia, smoking, alcohol abuse, obesity), chronic renal insufficiency, ischemic heart disease, peripheral vascular disease, atrial fibrillation, valvular heart disease, other cardioembolic sources; 3) Pre-treatment with antiplatelet agents; 4) The CHA_2_DS_2_Vasc score and the HASBLED score; 5) Statin status: pre-treatment or no pre-treatment with any statin. In patients receiving statins, the specific statin compound and its dose were recorded. In addition, the intensity of the statin therapy was classified into 3 types (low, medium or high) according to accepted guidelines^14^.

### Neuroradiological evaluation

MR scans were performed according to standard protocols at each site and at least included a T2*-weighted gradient-recalled echo (T2*-GRE) and/or susceptibility-weighted imaging (SWI) for the assessment of MB and cortical SS, and fluid-attenuated inversion recovery (FLAIR) for the assessment of LK. Two neuroradiologists, who were blind to clinical data including the statin status, evaluated the MR scans. The following abnormalities were noted: 1) MB, defined as rounded small lesions of less than 10 mm, as evidenced in echo-gradient or susceptibility images after ruling out MB mimics. In addition, its distribution (hemispheric -cortical, deep, or both) were recorded; 2) LK, defined as deep and periventricular white matter hyperintense lesions detected on T2 or FLAIR sequences. Its severity was quantified by the Fazekas’ scale^15^; 3) Cortical SS, defined as signal loss on T2*-GRE and SWI sequences in a curvilinear pattern following the gyral cortical surface^16^.

### Statistical analysis

We compared demographic, clinical and neuroimaging variables of patients with or without prior treatment with statins. The results are expressed as percentages for categorical variables and as mean and standard deviation (SD) for the continuous variables. The proportions were compared using the Chi-square (χ^2^) test or the Fisher exact test when it was required, while the mean and SD of continuous variables between groups were compared by the Student’s t-test. Median values for CHA_2_DS_2_Vasc and HASBLED scores were compared by the Mann-Whitney U test. We repeated the bivariate analyses by dividing the patients into four groups (no prior treatment, and low, medium or high prior intensity treatment). We also analyzed separately also each of the specific statin compounds. Finally, we performed a logistic regression analysis, to investigate the variables associated with MB. This was a forward stepwise analysis with those variables with a p ≤ 0.1 in the bivariate analyses. We adjusted the final model for age and potential confounders.

### Data availability

The datasets generated during and/or analysed during the current study are available from the corresponding author on reasonable request.

## Results

We studied 470 patients. Their mean age was 77.5 ± 6.4 years and 43.7% of them were men. The reason to prescribe OA was AF for 418 patients, atrial flutter for 15, valve disease for 9, dilated myocardiopathy for 6 and other reasons for 23. For the substudy of statin compound and dosage we excluded 15 patients in whom this information was unknown. A total of 193 (41.1%) patients were receiving statins prior to admission and the specific statin that was administered and its dose details are listed in Table 1. The intensity of statin treatment of the 178 patients with known dose was as follows: 28 patients (15.7% of the statin group) received a low dose, 118 (66.2% of the statin group) received a medium dose and 32 patients (17.9% of the statin group) received a high dose. Most of the patients who received statins prior to their stroke were treated with atorvastatin (n = 76) or simvastatin (n = 80).

**Table 1:** Characteristics of the statin therapy: statin and daily dose.

Name brand and daily dose of statin	Number (%)	Intensity
Simvastatin	80 (17%)	
10 mg	15	Low
20 mg	46	Medium
40 mg	15	Medium
Dose unknown	4	N/A
Atorvastatin	76 (16.2%)	
10 mg	12	Medium
20 mg	32	Medium
40 mg	21	High
80 mg	9	High
Dose unknown	2	N/A
Pravastatin	10 (2.1%)	
10 mg	1	Low
20 mg	7	Low
40 mg	2	Medium
Pitavastatin	1 (0.2%)	
1 mg	1	Low
Lovastatin	4 (0.8%)	
10 mg	1	Low
20 mg	3	Low
Rosuvastatin	10 (2.1%)	
5 mg	2	Medium
10 mg	6	Medium
20 mg	2	High
Fluvastatin	3 (0.6%)	
80 mg	3	Medium
Treatment with statin with specific compound unknown	9 (2%)	N/A

Values are given in number and (percentage).

Table 2 shows the demographic data of patients with and without prior statin treatment. As expected, patients in the statin group had a higher frequency of vascular risk factors: hypercholesterolemia, hypertryglyceridemia, diabetes, hypertension and of previous ischemic events in the peripheral, coronary or cerebral areas. Also, they had higher scores on the CHA_2_DS_2_Vasc and HAS-BLED scores and a higher frequency of concomitant treatment with antiplatelet agents.

**Table 2:** Demographic data, vascular risk factors, risk scores, and MR results in patients with or without prior statin therapy.

Variable	Prior statin treatment (n = 193)	No prior statin treatment (n = 277)	p value
Age (y)	78.01 ± 5.5	77.2 ± 7	0.21
Sex distribution (% men)	44	43.3	0.88
Type of stroke (% cerebral infarct)	84.9	88.4	0.17
Previous cerebral infarct	26.9	19.1	0.05
Previous Transient Ischemic Attack	14	5.4	0.002
Previous Intracerebral Hemorrhage	0.5	1.4	0.34
Hypertension	75.6	65.3	0.007
Diabetes Mellitus	26.9	13	<0.001
Hypercholesterolemia	79.8	19.5	<0.001
Hypertriglyceridemia	10.4	4	0.002
Smoking	7.8	7.6	0.65
Alcohol abuse	3.6	4	0.76
Obesity	21.8	15.5	0.13
Chronic renal failure	7.8	6.1	0.49
Ischemic heart disease	24.4	5.4	<0.001
Peripheral vascular disease	8.3	1.4	0.001
Previous Atrial Fibrillation	48.7	48.4	0.36
Valvular heart disease	3.1	4	0.59
Other cardioembolic sources	4.1	3.6	0.62
Prior treatment with anti platelets	61.1	30.7	<0.001
CHA_2_DS_2_Vasc score [median (IQ range)]	6 (5–6)	5 (4–6)	<0.001
HASBLED score [median (IQ range)]	2 (2–2)	2 (2–2)	0.007
Microbleed (yes or not)	30.6	29.2	0.76
Microbleed burden			
0	69.4	70.4	
1	18.7	13.7	0.24
>1	11.9	15.5	
Microbleed distribution hemispheric			
lobar	12	6.9	
deep	13.5	18.4	0.14
both	5.2	4	
Cortical siderosis (yes or not)	2.7	5.5	0.17
Leukoaraiosis			
0	18	17.1	
1	34.9	37.8	0.17
2	22.8	28.4	
3	24.3	16.7	

Values are given in percentage, unless specified otherwise.

MR images were obtained with a 1 T file strength in 40 (8.5%) patients, 1.5 T in 365 (77.8%), and 3 T in 64 (13.6%) patients. T2*-GRE sequences only were obtained in 187 patients, SWI only in 4 and both in 278. MB were detected in 140 (29.7%) patients. The distribution was lobar in 55%, deep in 30% and both in 15% of the patients. A total of 66 of them (47% of the MB group and 14% of the total patients) had more than one MB, and 8 (6% of the MB group and 1.7% of the total sample) more than 10 MBs. There were no differences in the presence of MB or in the MB burden between those patients with and without statin treatment. Moreover, the distribution of MB was not different between patients who were pre-treated or those who were not pre-treated with statins (Table 2). LK was detected in 388 (82.5%) of the patients and was severe in 19.8%. Cortical SS was detected in 20 (4.3%) patients. Likewise, the presence of cortical SS and the presence and burden of LK were equivalent in patients with and without previous statin treatment. As shown in Table 3, the presence or burden of MB, presence and degree of LK and presence of cortical SS according to the statin intensity also showed no differences among statin intensity groups. The results were equivalent for treatment with atorvastatin and simvastatin.

**Table 3:** Surrogate markers of angiopathy according to the intensity of statin pre-treatment.

	Low intensity (n = 28)	Medium intensity (n = 118)	High intensity n = 32	p value
MB	12 (42.9)	33 (28)	9 (28.1)	0.29
MB burden				
0	16 (57.1)	85 (72)	23 (71.9)	
1	7 (25)	20 (16.9)	4 (12.5)	0.53
>1	5 (17.9)	13 (11)	5 (15.6)	
MB distribution (n = 54 with 1 or more MB)				
Lobar	5 (41.7)	14 (42.4)	3 (33.3)	
Deep	5 (41.7)	14 (42.4)	3 (33.3)	0.81
Both	2 (16.7)	5 (15.2)	3 (33.3)	
Cortical superficial siderosis	1 (4)	4 (3.6)	0 (0)	0.54
Leukoaraiosis score				
0	5 (18.5)	18 (15.7)	7 (21.9)	
1	11 (40.7)	42 (36.5)	7 (21.9)	0.66
2	6 (22.2)	27 (23.5)	7 (21.9)	
3	5 (18.5)	28 (24.3)	11 (34.4)	

Values are given in number and percentage.

Finally, we performed a logistic regression analysis to predict the presence of MB. From a first model including those variables that in the bivariate analyses (Table 4) showed an association (p ≤ 0.1) with the risk of finding MB (previous cerebral infarct, previous intracerebral hemorrhage, smoking, alcohol abuse, obesity, hypertension and previous antiplatelet treatment) we conducted a forward stepwise selection method (Table 5). The model was adjusted by known potential contributors. When adjusting the final model for age, prior ICH (OR 9.70, 95% CI 1.066–90.9) and prior antiplatelet (OR 1.66, 95% CI 1.09–2.53) remained the only variables associated with the risk of MB. Remarkably, the inclusion of the variable prior statin therapy did not alter the final model coefficients and did not show any interaction with the variables included.

**Table 4:** Bivariate analyses of variables in patients with and without MB.

Variable	MB yes (n = 140)	MB no (n = 330)	p value
Age (y)	78 (6.9)	77.3 (6.2)	0.34
Gender distribution (% men)	45	43	0.88
Previous cerebral infarct	25.7	20.9	0.058
Previous Intracerebral Hemorrhage	2.9	0.3	0.006
Hypertension	68.5	72.1	0.097
Diabetes Mellitus	19.3	18.5	0.25
Hypercholesterolemia	42.9	44.8	0.211
Hypertriglyceridemia	7.1	6.4	0.34
Smoking	10	6.7	0.083
Alcohol abuse	1.4	4.8	0.067
Obesity	19.3	17.6	0.078
Chronic renal failure	8.6	6.1	0.11
Ischemic heart disease	12.9	13.3	0.211
Peripheral vascular disease	5.7	3.6	0.203
Previous Atrial Fibrillation	51.4	47.3	0.175
Prior treatment with antiplatelets	50.7	40	0.015
Prior treatment with statins	42.1	40.6	0.760
CHA_2_DS_2_Vasc score [median (IQ range)]	5 (4–6)	5 (4–6)	0.96
HASBLED score [median (IQ range)]	2 (2–2)	2 (2–2)	0.324

Values are given in percentage, unless specified otherwise.

**Table 5:** Logistic regresion analysis to predict the presence of MB.

	Variables	OR	95% IC	p
Model 1*	Previous ICH	9.708	1.057–90.909	0.044
	Previous antiplatelet	1.694	1.116–2.570	0.013
Model 2^†^	Previous ICH	9.708	1.066–90.909	0.044
	Previous antiplatelet	1.669	1.098–2.538	0.016

*(Adjusted for prior cerebral infarct, smoking, alcohol abuse, obesity and arterial hypertension).

^†^(Adjusted for age).

## Discussion

Our multicenter observational study of patients with cardioembolic stroke indicated that patients who received statins prior to stroke did not show a higher frequency or burden of surrogate MR markers for bleeding-prone angiopathies than patients who did not receive statins. This suggests that statins do not interact with hemorrhagic-prone small-vessel angiopathies. Also, we found that the intensity of the statin treatment was not associated with the presence or the burden of MB, LK or cortical SS.

The reason why there is an association between statin treatment and an increased risk of intracranial hemorrhage (ICH) is not fully understood. Some previous studies suggested that statin therapy increases the risk of ICH, specially in patients with a history of cerebrovascular disease^2,17^. In patients treated with statins there is an increase in symptomatic intracerebral hemorrhage after intravenous or endovascular reperfussion therapies^18–21^. However, this increased risk of ICH after exposure to statins has not been confirmed by other trials or meta-analyses^22–25^. Some explanations have been presented for the increased hemorrhagic risk associated with statin therapy. First, statin therapy is antithrombotic^3^, and it enhances anticoagulant and antiplatelet effects as well as an enhanced fibrinolysis. Second, statin therapy lowers cholesterol levels. Cholesterol levels are inversely associated with risk of hemorrhagic stroke^26–29^. It is important to note that LDL levels at baseline and during treatment were not related to haemorrhage risk in the SPARCL^30^ and PCSK9 inhibitors trials^31,32^.

Whether statin increases the risk of microhemorrhages, evidenced by MB, or other hemorrhagic-prone angiopathy markers is unknown. MB is a marker of hypertensive and amyloid angiopathies, which are the most frequent etiologies in patients with spontaneous ICH^33,34^. By analogy with OA and antiplatelet treatment, it is plausible that the antithrombotic properties of statins might increase the bleeding risk of macro as well as microhemorrhages. Whereas some authors advise to avoid statin therapy after an ICH, whether this is prudent also when micro- instead of macrohemorrhages are detected is unclear^35^. In our multicenter study of 470 patients, 41% of our patients were taking statins before stroke, but we were unable to find an association between statin treatment and MB. About 30% of our patients exhibited at least 1 MB, 4.5% cortical SS and 80% some degree of LK. We restricted our study to patients with cardioembolic stroke because, although they constitute the subtype of ischemic stroke with less MB, they often are prescribed OA^36^. We included only patients who were new users of OA to avoid any possible influence of previous treatment with OA in the development of these markers^37^. Moreover, although the concomitant treatment with antiplatelets was related in our study to the presence of MB, the use of statins was not, even after including this variable in our logistic regression analysis.

We evaluated different intensities of statin therapy and found that there was no effect on MB, LK, and cortical SS^14^. Finally, due to the reported differences and even opposite effects among statin compounds we investigated the different effects of the two most prescribed statin brands (simvastatin and atorvastatin)^38^. We did not find any influence on the presence or burden of hemorrhagic-prone markers.

Our results are in accordance with two studies^6,7^. One retrospective study of patients with ischemic stroke was unable to find any association between statin treatment and MB in any location^6^. Importantly, the etiologic stroke subtype was not analyzed in this study. A 2-year follow-up study of patients with stroke of any etiology included in the VITATOPS trial demonstrated also that statin treatment had no effect on incident lacunes or incident MB^7^.

Conversely, other studies suggest that statins might cause cerebral amyloid angiopathy (CAA) or MB, and may therefore trigger bleeding complications, but it is unknown whether statin treatment or low colesterol levels increase the amount of MB^10^. A recent study reported that the protective effect of serum cholesterol against intracerebral hemorrhage was reduced by statins but only in lobar regions^8^. The Framingham Heart Study indicated that statin increased the risk of lobar and mixed location MB^4^. A multivariate analysis of patients with or without stroke found an association between very low total cholesterol levels and MB burden^9^. Finally, a retrospective multivariate analysis of patients with intracerebral hemorrhage found that patients who were treated with statins had almost twice as many cortical MB compared to patients not treated with statins^5^. Unfortunately, these studies are not completely comparable with our study since they include patients without stroke or with hemorrhagic stroke^4,5,8,9^. Because we found that lobar MB and cortical SS are not related to previous statin therapy, we propose that statins do noy play a role in the development of intracranial angiopathies. Further studies, including genetic analyses, are needed to clarify this point^10,39^. There may be a subset of patients with certain genetic traits (such as ε2 and ε4 alleles of the Apolipoprotein E gene) that may confer susceptibility to brain microbleeds after statin exposure.

Our study has some limitations. First, we cannot be sure that the prescription of OA toghether with statins during secondary prevention is safe. The results of ongoing studies may help clarifying whether statins are safe in patients who have suffered a cardioembolic ischemic stroke and receive OA. No interaction between statin treatment and antithrombotic treatments were detected in the SPARCL study, and a small randomized trial found no increase in the risk of bleeding in patients who received statin and OA^30,40^. We have not provided information on statin adherence and in duration of previous treatment. It is possible that harmful effects of statin, if any, only appear after a long period of treatment. Moreover, the non-randomized use of statins in our study means that statin use is confounded by the indication for statin prescription. We did not measure colesterol levels and therefore cannot affirm or deny that they are associated with the markers. Statin treatment with high doses, a surrogate for lower levels of cholesterol, argues against the relevance of colesterol levels in the development of MB. We measured the radiological markers from different centers that used different imaging protocols and different MR apparatus of different field strength, and this could affect our results. Finally, the statistical power may not be sufficient to analyse the impact of statins on a number of surrogate markers.

## Summary

Statin therapy does not appear to increase the risk of MB, cortical SS or LK in patients with cardioembolic stroke who have not received OA. Our results suggest that the potential mechanism of statin-associated ICH, is not mediated by an increase in MB, cortical SS or LK. However, further studies including follow-up data after OA are needed. In the absence of definitive results, it is prudent to not discontinue or withhold statin treatment when MB, cortical SS or LK are detected.
